# The Role of Liver Fibrosis Assessment in the Management of Patients with Chronic Hepatitis B Infection: Lessons Learned from a Single Centre Experience

**DOI:** 10.1155/2011/524027

**Published:** 2011-10-30

**Authors:** Raza Malik, Patrick Kennedy, Deepak Suri, Ashley Brown, Rob Goldin, Janice Main, Howard Thomas, Mark Thursz

**Affiliations:** ^1^Liver Center, Department of Medicine, Beth Israel Deaconess Medical Center, Harvard Medical School, 110 Francis Street, Suite 4A, Boston, MA 02215, USA; ^2^Institute of Cell and Molecular Science, Barts and The London School of Medicine, 4 Newark Sreet, London E1 4AT, UK; ^3^Hepatology Unit, Division of Gastroenterology, University College London Hospital, 235 Euston Road, London NW1 2BU, UK; ^4^Hepatology Unit, Department of Medicine, 10th Floor, St Mary's Hospital, Imperial College London, Praed Street, London, W2 1NY, UK

## Abstract

*Background & Aims*. Assess the clinical utility of the Prati criteria and normal ALT (<40 IU/L) in a cohort of patients with chronic hepatitis B infection (CHB). *Methods*. Serology, radiology, and histology were obtained in 140 patients with CHB. *Results*. HBeAg^+^ group: 7 patients (7/56−12% HBeAg^+^ group) misclassified as “immunotolerant”, with HBV DNA > 6 log copies/ml and normal ALT, who in fact had moderate/severe fibrosis on liver biopsy. HBeAg^−^ group: 10 patients with normal ALT and moderate/severe fibrosis on liver biopsy; 4 of these patients had >3 log copies/ml HBV DNA levels and 6 patients misclassified as “inactive carriers” with negative HBV DNA levels normal ALT and moderate/severe fibrosis (6/84−7% HBeAg^−^ group). Two male HBeAg^+^ and three male HBeAg^−^ patients with ALT between 20 and 30 IU/L and moderate/severe fibrosis on liver biopsy would have been further mischaracterised using the Prati criteria for normal ALT. Age and ethnic group were more important predictors of moderate/severe fibrosis in multivariate analysis. *Conclusion*. HBeAg status, age, ethnic origin with longitudinal assessment of LFTs and viral load should be studied in patients with “normal ALT” at the upper end of normal range (ALT 20–40 IU/L) to appropriately classify patients and identify patients for liver fibrosis assessment to inform treatment decisions.

## 1. Introduction

An estimated 400 million people worldwide have chronic hepatitis B virus infection, of whom 1 million people will die each year from its complications [1, 2]. In the United Kingdom, it is estimated that over 180,000 people are infected, with the numbers increasing rapidly (http://www.hepb.org.uk). 

In general, patients with chronic hepatitis B infection have historically been stratified into 4 classic categories according to the natural history of the infection [2, 3].

1. Immune-tolerant phase: HBeAg positive with high levels of viral replication (HBV DNA levels: >6 log), normal ALT, without necroinflammation in the liver and a low risk of progression to cirrhosis.
2. Immune-active phase: HBeAg positive with high levels of viral replication (HBV DNA levels: >6 log), increased ALT, necroinflammation in the liver with a high risk of progression to cirrhosis.
3. Inactive HBV carrier state: these patients have undergone seroconversion to anti-HBe status. It is characterised by very low/undetectable HBV DNA levels, normal ALT, without necroinflammation on liver histology and low risk of progression to cirrhosis.
4. HBeAg-negative chronic hepatitis B infection (precore/basal core mutant disease) the development of a pre-core/basal core mutant results in active viral replication. These patients have detectable HBV DNA levels (but traditionally lower than HBeAg-positive patients), with fluctuating levels of ALT, necroinflammation in the liver, and a high risk of progression to cirrhosis.

The European Association for the Study of the Liver (EASL) and the American Association for the Study of Liver Diseases (AASLD) have both recently published guidelines on the management and treatment of chronic HBV [3–5]. Before embarking on a treatment algorithm, it is important to identify the parameters that determine which patients require treatment [6]. These parameters as recommended by the Asian Pacific Association for the Study of the Liver (APASL), EASL, and AASLD include hepatitis serology, HBV DNA levels, serum ALT, and liver histology. Recent recognition of the important prognostic value of HBV DNA levels is reflected in the removal of emphasis on liver biopsy in the assessment of patients with chronic hepatitis B infection [7]. Current guidelines recommend selection of patients for liver biopsy based primarily on their ALT values. Until recently a so-called “normal ALT” (ALT < 40 IU/L) was thought to exclude hepatic inflammation and significant liver fibrosis. However, recent evidence in chronic hepatitis C infection suggests that a normal ALT at the upper limit of normal contains a significant number of patients with hepatic inflammation and liver fibrosis on liver biopsy [8, 9]. This effect has also been shown in other liver diseases resulting in the development of the Prati criteria for normal ALT in the last decade. The Prati criteria for normal ALT is an ALT < 30 IU/L in men and ALT < 19 IU/L in women [10, 11].

The aim of the study was to perform a cross-sectional analysis of a population of patients with chronic hepatitis B infection using serological, radiological, and histological data to assess the clinical utility of a normal ALT (<40 IU/L) and the Prati criteria.

## 2. Method

### 2.1. Patient Selection

Adult patients with chronic HBV infection (defined as HBsAg-positive for greater than 6 months) were recruited from primary care, antenatal, or sexual health clinics at a single centre at St Mary's Hospital, London, UK. The study was approved by the hospital ethics committee, with a total of 140 treatment naive chronic HBV patients recruited into the study between 2003 and 2008. The patients were recruited prospectively in a consecutive manner. The patients had the following assessment.

### 2.2. Clinical Assessment

A complete history and physical examination was performed on all patients. Patients with a high alcohol intake (>150 grams/week) were excluded from the study.

### 2.3. Serum Analysis

A serum sample was obtained from each patient at the initial study visit as part of the IRB protocol. Laboratory investigations included routine CBC, serum electrolytes, and liver biochemistry (ALT, AST, bilirubin, ALP, GGT). Hepatitis B serology included HBsAg/Ab, HBeAg/Ab, and HBcAb using standard assays. Serum HBV DNA (copies/mL) was measured using ABBOTT real-time quantitative PCR and ABI PRISM.

### 2.4. Radiology Tests

All patients had a liver ultrasound scan. The scans were reported by a consultant radiologist with specialist expertise in liver radiology.

### 2.5. Liver Biopsy and Histopathology

The histological diagnosis was established using hematoxylin & eosin (H & E) staining and Masson's trichrome stains of formalin fixed paraffin-embedded liver tissue. Two specialist liver histopathologists reviewed all the biopsy material and formally allocated fibrosis scores to each specimen based on a modified Ishak scoring system. Patients with a fibrosis stage of 0–2 were designated as having mild disease, 3-4 moderate disease, and 5-6 severe disease (6 = cirrhosis). Necro-inflammatory scores of 0–3 indicated mild inflammation whilst a score > 3 was considered significant inflammation.

### 2.6. Patient Cohorts and Study Design

A combination of hepatitis serology, HBV DNA levels, serum ALT, and liver histology was obtained in a total of 140 patients with chronic hepatitis B infection: HBeAg^+^ (*n* = 56) and HBeAg^−^ (*n* = 84). The serum analysis, radiology, and liver biopsy were all performed within 3 months of each other, with a median time delay of 23 days between the corresponding tests in each patient. The data was collected prospectively and the analysis performed retrospectively.

### 2.7. Statistical Analysis

Numerical data are expressed as mean +/−  standard deviation. The *t*-test was used to compare paired data. 

Regression analysis was performed using SPSS software. Univariate analysis identified an association between fibrosis and a variety of patient variables. Multivariate analysis was performed on factors found to be significant (*P* < 0.05) on univariate analysis, to identify predictors of fibrosis, and *P*-values were generated for each of the variables.

## 3. Results

There were a total of 140 patients recruited into the study, with the demographics and characteristics of the patients provided in Table 1. The prevalence of hepatic steatosis on liver in USA was 8% (11 patients) in the study. All these patients had an abnormal ALT (ALT > 40 IU/L). In addition, 5 of the 11 individuals with hepatic steatosis had a serum ALT > X2 ULN (>80 IU/L) and NASH on liver biopsy (macrovesicular steatosis, ballooning degeneration, and lobular inflammation) complicating the hepatitis B infection. 

The HBeAg^+^ patients (*n* = 56) and HBeAg^−^ patients (*n* = 84) were stratified according to serum ALT. In the HBeAg^+^ cohort, all the ALT subgroups had high levels of HBV DNA (>6 log copies/mL) as would be expected for HBeAg^+^ patients (Tables 2 and 3). In the HBeAg^−^ patients, there was a variation in HBV DNA levels within the different ALT subgroups, with the higher ALT group X2 ULN (>80 IU/L) consisting of more patients with high HBV DNA levels, as a result of the emergence of precore mutant disease (Table 3). As expected, in both HBeAg^+^ and HBeAg^−^ cohorts, the patients with high ALT levels had higher necroinflammatory scores (*P* < 0.05) and fibrosis scores (*P* < 0.05) than patients in the lower ALT subgroups (Tables 2 and 3). 

The so-called “immunotolerant” patients in the HBeAg^+^ cohort and the “inactive carriers” in the HBeAg^−^ cohort were further scrutinized. In the HBeAg^+^ group, there were a total of 25 patients with normal ALT (<40 IU/L). There were 7 patients (7/56−12% of total HBeAg^+^ group) misclassified as “immunotolerant”, with HBV DNA > 6 log copies/mL and normal ALT, who in fact had moderate/severe fibrosis on liver biopsy (Table 2). Statistical analysis showed that an abnormal ALT (ALT > 40 IU/L) yielded a sensitivity of 72% and specificity of only 58% as a predictor of moderate/severe liver fibrosis in the HBeAg^+^ group (Table 4). 

In the HBeAg^−^ group, there were a total of 29 patients with normal ALT (<40 IU/L). There were 10 patients with normal ALT and moderate/severe fibrosis on liver biopsy; 4 of these patients had >3 log copies/mL HBV DNA levels and 6 patients were misclassified as “inactive carriers” with negative HBV DNA levels and normal ALT (6/84−7% of total HBeAg^−^ group) (Table 3). Statistical analysis showed that an abnormal ALT (ALT >  40 IU/L) yielded a sensitivity of 85% and specificity of only 53% as a predictor of moderate/severe liver fibrosis in the HBeAg^−^ group. 

In total, 9% of patients in the study (13/140; 7 eAg^+^ and 6 eAg^−^) with moderate/severe fibrosis would have been incorrectly classified as immune tolerant or inactive carriers if liver biopsy had not been performed. Subgroup analysis of males in our study with ALT 20 and 30 IU/L is also important in relation to the application of the Prati criteria for normal ALT. There were two male (immunotolerant) HBeAg^+^ and three male (inactive carrier) HBeAg^−^ patients with ALT between 20 and 30 IU/L and moderate/severe fibrosis on liver biopsy, who would have been mischaracterised as normal if using the Prati criteria for normal ALT (ALT < 30 IU/L in men and ALT < 19 IU/L in women).

It is noteworthy that two patients in the HBeAg^+^ cohort with normal ALT were cirrhotic and one patient in the HBeAg^−^ cohort had negative HBV DNA levels (<3 log copies/mL), normal ALT and cirrhosis on liver biopsy emphasising further the importance of appropriately stratifying patients with upper limit normal serum ALT.

Regardless of ALT not being a good predictor of liver fibrosis in this study, an important additional finding was the ability of the inflammatory score on liver biopsy to correlate well with the serum ALT. Hence patients with ALT < 20 IU/L had much lower NI scores than patients with ALT 20–30 or above (Tables 2 and 3).

### 3.1. Risk Factors and Correlation with Liver Fibrosis in Patients with Normal ALT

Factors that determined moderate/severe liver fibrosis were sought in all the chronic HBV patients. The effects of age (>45), gender (male), ethnic group (Asian/African), HBV DNA level (>6 log copies/mL), serum ALT level (ALT > 40), and necroinflammatory score (N.I. > 3) at the time of liver biopsy were analysed in regression analysis using SPSS software. In univariate analysis, there was a significant correlation with age, ethnic group, HBV DNA levels, and necroinflammatory score (*P* < 0.05). In multivariate analysis, only patient age and ethnic group were associated with moderate/severe liver fibrosis (Table 3— *P* < 0.05).

## 4. Discussion

This chronic hepatitis B infection study was designed as a cross-sectional study assessing serological, radiological, and histological data as is frequently encountered in clinical practise. The results of this study are important as they show that classification and stratification of patients into immunotolerant and inactive carriers can be difficult with a single blood test using hepatitis B serology, viral load, and liver function tests. The study results show that a single serum ALT alone can give a false impression and be misleading when predicting advanced liver disease in a small proportion of patients, even if using the Prati criteria for normal ALT (ALT < 30 IU/L in men and ALT < 19 IU/L in women). Given the large number of patients worldwide with chronic hepatitis B infection (>400 million), the number of patients missed will be significant [12, 13]. 

The idea of a liver biopsy in all adult HBeAg^+^ and HBeAg^−^ patients with “normal ALT” at the upper end of normal range (ALT 20–40 IU/L) is a daunting prospect for clinicians as it goes beyond the Prati criteria, and liver biopsy has well-recognised complications as well as cost implications. The limitations of liver biopsy include sampling error, interobserver variability in reporting and the small but significant morbidity and mortality associated with this invasive procedure [14, 15]. Hence a focused approach using HBeAg status, age, ethnic origin with longitudinal assessment of LFTs and viral load should be studied in patients with “normal ALT” at the upper end of normal range (ALT 20–40 IU/L) to appropriately classify patients and select patients that require an assessment of liver fibrosis to inform treatment decisions. The liver fibrosis assessment in selected patients could utilise novel noninvasive technologies, which have the potential to identify liver fibrosis in patients with chronic hepatitis B infection. Serum markers of liver fibrosis and transient elastography could be performed in selected patients with “normal ALT” at the upper end of normal range (ALT 20–40 IU/L), to identify the small proportion of patients with moderate/severe liver fibrosis [16–19]. An abnormal result could trigger a liver biopsy and thereby reduce the risk from an invasive procedure in a significant number of patients, whilst also providing the required information for OAV treatment decisions [20–22]. A further benefit with this approach is evident in subgroup analysis of males in our study with ALT 20–30 IU/L. There were two male HBeAg^+^ and three male HBeAg^−^ patients with ALT between 20–30 IU/L who had moderate/severe liver fibrosis on biopsy. These individuals fall within the normal range by the Prati criteria and, therefore, would have been missed if the Prati criteria alone was used as an indication for liver biopsy.

A major limitation of this study is based on its cross-sectional design. There will be fluctuations in serum ALT and HBV DNA missed in cross-sectional analysis and patient misclassified as immune tolerant or inactive carriers. If patients were followed longitudinally with serial blood tests including HBV viral load and LFTs, then appropriate classification would be achieved. Hence, the standard of care should be serial blood tests to appropriately classify patients as long as the time delay does not result in progression in liver disease or hepatic decompensation [13].

This study shows a correlation between serum ALT and necroinflammatory score as would be expected (in cross-sectional analysis) as inflammation is immediately reflected; however, fibrosis takes much longer to establish, hence serum ALT bears no relation to liver fibrosis in the study.

In conclusion, HBV infection is a chronic, dynamic infection that evolves over many years with both viral and host factors determining disease activity [2]. This study indicates that HBeAg status, age, ethnic origin with longitudinal assessment of LFTs, and viral load should be studied in patients with “normal ALT” at the upper end of normal range (ALT 20–40 IU/L) to appropriately classify patients and identify patients for liver fibrosis assessment to inform treatment decisions.

## Figures and Tables

**Table 1:** Demographics and characteristics of the chronic Hepatitis B infection cohort.

Variable	Outcome
Total (*n*)	140
Age (years)	39.5 ± 13.4
Gender (male)	84 (60%)
eAg status (+'ve)	56 (40%)
Ethnic origin (C, A, AC)	76 (54%), 45 (32%), 19 (14%)
Serum ALT (IU/L)	86.6 ± 53
Platelet count	Normal (245 ± 23)
Serum bilirubin, INR, Albumin	normal (15 *μ*mol/L), normal (1), normal (40 g/L)
Liver biopsy length	3.4 ± 1.1 cm
Necroinflammatory score (Ishak)	4.0 ± 2
Fibrosis score	2.8 ± 1.8

Results are expressed as mean ± standard deviation and percentages.

Ethnic origin (C: Caucasian; A: Asian; AC: Afro-Caribean).

**Table 2:** The characteristics of 56 HBeAg^+^ patients stratified according to serum ALT.

ALT category	Total number of patients	HBV DNA (copies/mL)	NI Score	Mild fibrosis (no. of patients)	Mod/Severe fibrosis (no. of patients)
<20	8	(>6 Log)	1± 1.5	8	0
20–30	6	(>6 Log)	2.4 ± 2.1	4	2
31–40	11	(>6 Log)	3.7 ± 2.3	6	5
41–80	16	(>6 Log)	4 ± 2.5	8	8
>80	15	(>6 Log)	5 ± 2.5	5	10

Results are expressed as mean ± standard deviation.

**Table 3:** Shows the characteristics of 84 HBeAg^−^ patients stratified according to serum ALT.

ALT category	Total number of patients	HBV DNA		NI score (mean)	Mild fibrosis (no. patients)	Mod/Severe fibrosis (no. patients)
		log ranges copies/mL	(no. patients)			
<20	1	<3 log	1	1	1	0
		3 log–6 log	0			
		>6 log	0			
20–30	13	<3 log	12	3.0	10	3
		3 log–6 log	1	±1		
		>6 log	0			
31–40	15	<3 log	12	3.5	8	7
		3 log–6 log	3	±1.5		
		>6 log	0			
41–80	37	<3 log	18	3.8	14	23
		3 log–6 log	11	±2.5		
		>6 log	8			
>80	18	<3 log	5	5.5	6	12
		3 log–6 log	3	±1.5		
		>6 log	10			

Results are expressed as mean ± standard deviation.

**Table 4:** Multivariate analysis of factors associated with moderate/severe liver fibrosis in the chronic hepatitis B cohort.

Category	Variable	Univariate analysis significant factors (*P* < 0.05)	Multivariate analysis significant factors (*P* < 0.05)
Age	Years (>45)	Yes (*P* < 0.05)	Yes (0.045)
Gender	Male	No	No
Ethnic group	Asian/Afro-Caribean	Yes (*P* < 0.05)	Yes (0.02)
Viral load	HBV DNA level (>6 log)	Yes (*P* < 0.05)	No
Serum ALT	ALT (>40)	No	No
Hepatic inflammation	Necroinflammatory Score (N.I.>3)	Yes (*P* < 0.05)	No
